# KRAS and EGFR Mutations Differentially Alter ABC Drug Transporter Expression in Cisplatin-Resistant Non-Small Cell Lung Cancer

**DOI:** 10.3390/ijms22105384

**Published:** 2021-05-20

**Authors:** Luca Jaromi, Veronika Csongei, Monika Vesel, ElHusseiny Mohamed Mahmud Abdelwahab, Amina Soltani, Zsofia Torok, Gabor Smuk, Veronika Sarosi, Judit Erzsebet Pongracz

**Affiliations:** 1 Department of Pharmaceutical Biotechnology, Faculty of Pharmacy, University of Pecs, 2 Rokus Str, H-7624 Pecs, Hungary; jaromi.luca@pte.hu (L.J.); csongei.veronika@pte.hu (V.C.); monika.avdicevic@gmail.com (M.V.); elhusseiny.mohamed@pte.hu (E.M.M.A.); aminasoltani8@gmail.com (A.S.); torok.zsofia@pte.hu (Z.T.); 2 Wnt-Signalling and Biotechnology Research Group, Szentagothai Research Centre, University of Pecs, 20 Ifjusag Str, H-7624 Pecs, Hungary; 3 Department of Pulmonology, Internal Medicine, The Medical School and Clinical Centre, University of Pecs, 12 Szigeti Str, H-7624 Pecs, Hungary; sarosi.veronika@pte.hu; 4 Department of Pathology, The Medical School and Clinical Centre, University of Pecs, 12 Szigeti Str, H-7624 Pecs, Hungary; smuk.gabor@pte.hu

**Keywords:** NSCLC, AC, KRAS, EGFR, ABC drug transporters, WNT signalling

## Abstract

Lung carcinoma is still the most common malignancy worldwide. One of the major subtypes of non-small cell lung cancer (NSCLC) is adenocarcinoma (AC). As driver mutations and hence therapies differ in AC subtypes, we theorized that the expression and function of ABC drug transporters important in multidrug resistance (MDR) would correlate with characteristic driver mutations KRAS or EGFR. Cisplatin resistance (CR) was generated in A549 (KRAS) and PC9 (EGFR) cell lines and gene expression was tested. In three-dimensional (3D) multicellular aggregate cultures, both ABCB1 and ABCG2 transporters, as well as the WNT microenvironment, were investigated. ABCB1 and ABCG2 gene expression levels were different in primary AC samples and correlated with specific driver mutations. The drug transporter expression pattern of parental A549 and PC9, as well as A549-CR and PC9-CR, cell lines differed. Increased mRNA levels of ABCB1 and ABCG2 were detected in A549-CR cells, compared to parental A549, while the trend observed in the case of PC9 cells was different. Dominant alterations were observed in LEF1, RHOU and DACT1 genes of the WNT signalling pathway in a mutation-dependent manner. The study confirmed that, in lung AC-s, KRAS and EGFR driver mutations differentially affect both drug transporter expression and the cisplatin-induced WNT signalling microenvironment.

## 1. Introduction

Lung carcinoma (LC) is still the most common malignancy and one of the leading causes of cancer-related death worldwide [1]. Despite significantly improved treatment modalities, five-year survival of LC barely exceeds 18% primarily due to late diagnosis [2,3,4]. The two main lung cancer types are small cell lung cancer (SCLC) and non-small cell lung cancer (NSCLC). Approximately 12 out of 100 LC patients are diagnosed with SCLCs (12%), while the larger proportion (88%) with NSCLCs. NSCLC has three common types: squamous cell carcinoma (SCC), adenocarcinoma (AC) and large cell carcinoma (LCC). Treatment of patients diagnosed with AC is based on specific marker mutations, including Kirsten rat sarcoma viral oncogene homologue (KRAS), epidermal growth factor receptor (EGFR), anaplastic lymphoma kinase (ALK), proto-oncogene 1, receptor tyrosine kinase (ROS1) and B-Raf Proto-Oncogene, Serine/Threonine Kinase (BRAF) mutations. The therapeutic approach depends on the presence or absence of targetable mutations. Patient samples are also tested for programmed death ligand-1 (PDL1) expression to determine suitability for immune-checkpoint intervention [5] especially if targetable mutations are not identified. Targetable therapies (e.g., the tyrosine kinase inhibitor (TKI) Erlotinib) [6,7] and the relatively slow-acting immune-checkpoint therapies (e.g., Nivolumab, Ipilimumab) are used in a rather small percentage of patients [8]. Although novel therapies are emerging [9], patients—especially at late or inoperable stages—are still treated by a set protocol involving surgery, radiation- or chemotherapy or a combination of the above.

Interestingly, the very heterogeneous AC has become the largest subpopulation of NSCLC in recent years [10]. The differences amongst AC subgroups are based on genetic and epigenetic alterations, which modify signal transduction pathways and metabolic processes [11,12]. The two most widespread lung AC mutations include KRAS and EGFR [13]. Interestingly, among non-smokers, EGFR mutations occur more frequently [14], while KRAS mutations are more characteristic for smokers [15]. Concomitant mutations of KRAS and EGFR genes are very rare and generally regarded as mutually exclusive, requiring differential therapeutic approaches. If patients carry EGFR mutations, targeted TKI-s are far more effective than other treatment modalities [16,17]. EGFR mutations are relatively rare (around 10%) in the Caucasian population, hence there are relatively few people that can benefit from such therapy [18]. Patients who are carriers of KRAS mutation have no significant response to TKI [16]. If their tumours are positive for PDL1 (50%<), they are eligible for immune checkpoint therapy [5].

Additionally, KRAS mutations lack the option for targeted therapy, and they are often negative for PDL1. Consequently, therapy usually begins with platinum-based drugs (cisplatin or carboplatin) [19,20]. Although platinum-based treatment can increase survival rates at all stages of the disease, MDR and consequent disease recurrence remain major issues [19,21,22]. The treatment, however, is rarely platinum-based monotherapy. Instead, paclitaxel, docetaxel, gemcitabine or vinorelbine are combined with cisplatin or carboplatin to increase treatment efficacy [23]. The tumour cells, however, actively work to clear the cell from medications and drug resistance and increased cellular survival occurs. The best studied drug transporters belong to the ATP-Binding Cassette (ABC) superfamily [24,25,26]. Inhibition of ABC transporters leading to entrapment of drugs in tumour cells is an important area in cancer research [27,28,29,30]. In a previous study, we showed that, if the two main subtypes of NSCLC, AC and SCC are exposed to acute cisplatin treatment, then they differentially activate the beta catenin-dependent WNT (Wingless-Type MMTV Integration Site Family) signalling pathway. Modulation of the beta-catenin dependent canonical WNT pathway activity leads to alteration in drug transporter expression, the process that is actively involved in chemo-resistance [30]. In the present work, we theorized that background mutations might also affect drug response and alter the expression of drug transporters within the two major lung AC subgroups. We also hypothesised that a differential mutation background can result in alterations in the WNT microenvironment. Such variances can influence cisplatin-induced alterations in the WNT signalling pathways and, as a result, affect drug transporter expression and, ultimately, change drug response to combination chemotherapy.

## 2. Results

### 2.1. Differential ABCB1 and ABCG2 mRNA Levels Correlate with Driver Mutations in Primary Human Lung AC Samples

**Figure 1:**
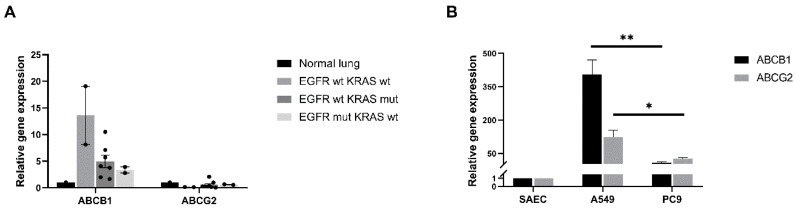
Drug transporter analysis of primary human adenocarcinoma (AC) samples and cell lines. (**A**) Relative mRNA expression (2^−ddCt^) levels of ABCB1 and ABCG2 drug transporters in adenocarcinoma patients (*n* = 12) with different mutational background (EGFR wild type/KRAS wild type; EGFR mutant/KRAS wild type, EGFR wild type/KRAS mutant). mRNA expression is relative to normal, healthy lung tissue. Data are presented as mean ± SD. (**B**) Relative mRNA expression (2^−ddCt^) levels of ABCB1 and ABCG2 in adenocarcinoma cell lines (A549, PC9). mRNA from primary human small airway epithelial cells (SAEC) was the control. The graph shows the mean and SD of biological repeats (*n* = 3), “*” *p* < 0.05 and “**” *p* < 0.002.

Twelve AC patient samples were tested for two drug transporters, ABCB1 and ABCG2. Both transporters are important in MDR, but no data are currently available about whether differences in background mutation would affect drug transporter mRNA levels. The samples represent the proportional distribution of KRAS and EGFR mutations among the Hungarian LC NSCLC patient population, and the small number of primary samples was used to select the appropriate cell lines for further studies. The non-treated primary LC samples were collected and grouped based on mutational background; then, qRT-PCR analysis was performed to detect ABCB1 and ABCG2 expression in the resected tumours (Figure 1A).

In general, in the primary tumours, the ABCB1 was the dominant transporter and the expression levels of ABCG2 was markedly lower. Commercially available normal, non-diseased lung mRNA (pooled from five individual donor samples) was used as a control. Patient samples were grouped as follows: where neither EGFR nor KRAS gene mutations were detected (WT), and where ABCB1 levels were very high, while ABCG2 mRNA levels were undetectable. In the EGFR mutant/KRAS WT and the KRAS mutant/EGFR WT patient groups both ABCB1 and ABCG2 were detected, although ABCB1 levels were higher in the presence of KRAS mutation. Although the sample pool was small, it indicated that approaching drug resistance from a mutation-specific angle might merit further investigation. As various manipulations are more difficult to perform on a limited number of primary patient samples, two lung AC cell lines, the KRAS mutant A549 and the EGFR mutant PC9 cell lines were selected for further experiments. In the case of the cell lines, commercially available, primary human small airway epithelial cells (SAEC) were used as controls. The tendency in ABCB1 and ABCG2 gene expression was higher than the normal SAEC control and had shown a similar tendency to the data in primary, untreated patient samples (Figure 1B). Generally, in the KRAS mutant A549 cells, both ABCB1 (*p* < 0.001) and ABCG2 (*p* < 0.035) gene expression levels were higher than in the EGFR mutant PC9 cell line (Figure 1B).

### 2.2. Cisplatin Resistance Alters ABC Transporter Expression and Function

**Figure 2:**
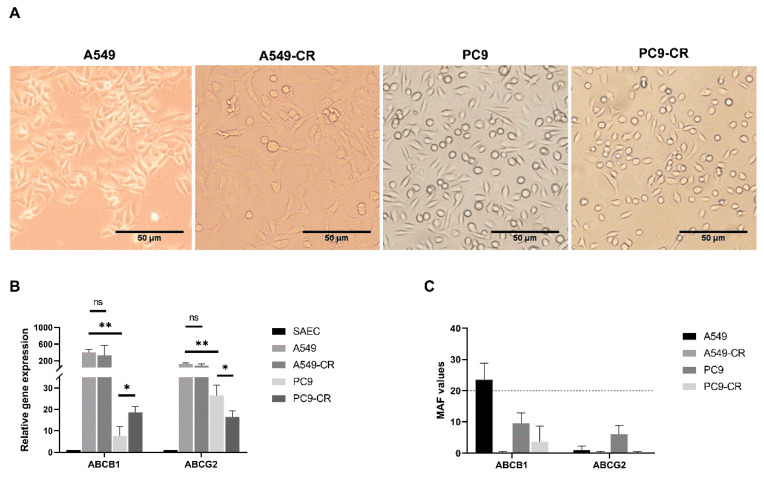
Adenocarcinoma morphology, drug transporter gene expression pattern and functional analysis of ABCB1, ABCG2 in 2D cultures. (**A**) Phase-contrast microscopic images (10×) from solvent control parental A549, cisplatin resistant A549 (A549-CR), solvent control parental PC9 and cisplatin resistant PC9 (PC9-CR). Scale bar is 50 µm. (**B**) Relative mRNA expression (2-ddCt) of ABCB1 and ABCG2 drug transporters in solvent control parental (A549, PC9) and cisplatin-resistant adenocarcinoma cell lines (A549-CR, PC9-CR) compared to normal, primary human SAEC. The graph shows the mean and SD of biological repeats (*n* = 3); “*” *p* < 0.05 and “**” *p* < 0.002. (**C**) Functional analysis of ABCB1 and ABCG2 drug transporters in control and cisplatin-treated (CR) A549 and PC9 cell lines, expressed as MAF values. Data presented as representatives of 4 independent measurements.

In our previous study [30], acute cisplatin treatment altered drug transporter expression via activation of the canonical WNT signalling pathway. Even the level of the ABC transporters which do not transport cisplatin were altered. We concluded at that time, that cisplatin treatment can serve as an inducer of MDR as apart from two cisplatin transporters, ABCC2 and ABCC6, cisplatin affects ABC transporters of several drugs [31,32,33]. To investigate the effect of chronic exposure to cisplatin in association with variations in mutation background, both A549 (KRAS mutant) and PC9 (EGFR mutant) lung AC cell lines received prolonged exposure to cisplatin at gradually increasing concentrations (A549 cell culture from 1–12 µM; PC9 cell culture from 0.67–4.67 µM) over a three-month period of time to achieve drug resistance. The treatment did not significantly change the morphology of the cisplatin resistant cells compared to parental controls, shown in Figure 2A.

The four cell lines were analysed for mRNA levels primarily for ABCB1 and ABCG2 (Figure 2B and Figure S1). ABCC1, ABCC2, ABCC6 and ABCC10 transporters that are important for cisplatin or other drugs, such as paclitaxel (ABCC10), used in combination therapy of lung AC are summarized in the supplementary data (Figure S1) [34,35,36,37,38]. First, the drug transporter mRNA expression levels of the two parental cell lines were compared to identify the mutational background on ABC transporter expression. Comparison of the parental cell lines A549 and PC9 showed that ABCB1 (*p* < 0.001) and ABCG2 (*p* < 0.035) genes were expressed significantly higher in the KRAS mutant A549 than in the EGFR mutant PC9 cells. As ABCC10 (*p* < 0.040) expression was significantly lower in A549 than in PC9 cells (Figure S1), the KRAS mutant AC-s are likely to be more sensitive to paclitaxel therapy than EGFR mutant lung AC-s.

In the next step, the effect of cisplatin was investigated on the two cell lines with different background mutations. There was no significant difference in the gene expression of the studied drug transporters in the parental control A549 and the A549-CR cells (Figure 2B). In contrast, in the EGFR mutant PC9 cell line ABCB1 (*p* < 0.021) transcript levels were at a significantly higher level in the PC9-CR than in the PC9 parental controls (Figure 2B). The absolute gene expression levels of both ABCB1 and ABCG2 transporters, however, remained significantly lower in the EGFR mutant PC9 cells than in the KRAS mutant A549 cells (Figure 2B). The significance of this finding is that EGFR mutation driven carcinogenesis might respond better to therapies sensitive to the above detailed drug transporters.

To investigate whether the two most prominent transporters were functionally active, we measured ABCB1 and ABCG2 protein activity using a commercially available kit. The activity of ABCB1 was higher in the parental control A549 cell line than in the A549-CR cells (Figure 2C). ABCG2 activity was undetectable in either the untreated control A549 or the drug resistant A549-CR cells. In the EGFR mutant PC9 parental and the drug resistant PC9-CR cells, neither ABCB1 nor ABCG2 activity was detectable (Figure 2C).

### 2.3. ABCB1 and ABCG2 Expression in 3D Tissue Aggregates of Cisplatin Resistant and Parental Controls of KRAS and EGFR Mutant Cell Lines

**Figure 3:**
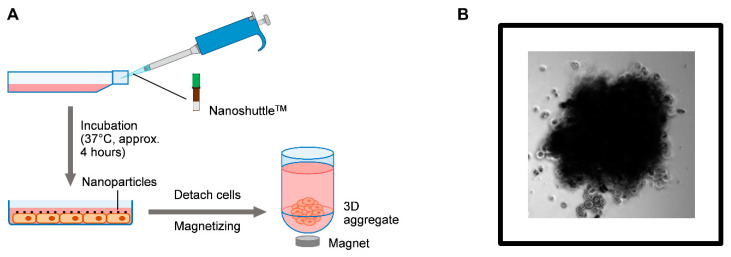
Formation of the 3D tissue aggregates. (**A**) Schematic representation of the preparation steps of 3D tissue aggregates using the Greiner Bio-One magnetization core technology with a NanoShuttle™-PL.-kit. (**B**) Light microscopic picture of a 3D PC9 tissue aggregate (20× magnification).

**Figure 4:**
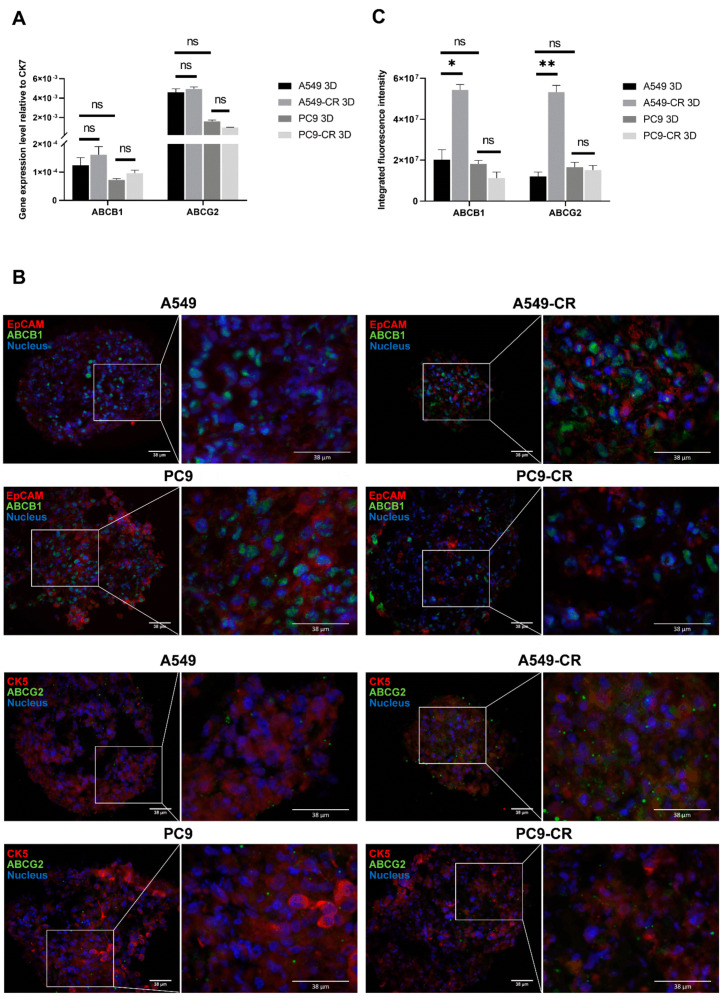
Mimicking cell composition of the human lung tissue. (**A**) Relative mRNA expression (2^−ddCt^) of ABCB1 and ABCG2 drug transporters in parental (A549, PC9) and cisplatin-resistant AC cell line (A549-CR, PC9-CR) containing 3D aggregate co-cultures. The inner control was CK7. Biological repeats *n* = 3. (**B**) 3D aggregate tissue sections were stained with anti-ABCB1 or anti-ABCG2 specific primary antibodies, respectively (green), while EpCAM and CK5 were stained with specific antibodies (red) and nuclei were stained with DAPI (blue). The staining shows a representative of 3 independent experiments. (**C**) Densitometry of ABCB1 and ABCG2 proteins in A549, A549-CR and PC9, PC9-CR containing aggregate co-cultures. Drug transporter protein intensity quantification was normalized to the respective epithelial marker intensity. (“*” *p* < 0.05 and “**” *p* < 0.002).

3D cell co-cultures are valuable models for testing tissue response to various drugs as cell–cell interactions, especially the presence of resident cell types increase similarities to the functional human tissue [39,40,41]. Drug transporter expressions are also more closely related to the levels in the normal human lung in 3D cultures [39,40,41,42]. To investigate whether there are any differences between the 3D multicellular (epithelial cancer cell line, endothelium, mesenchyme) aggregates, if the driver mutations are different, ABCB1 and ABCG2 expressions were tested in 3D co-cultures using KRAS and EGFR mutant parental and CR cell lines (Figure 3A,B and Figure 4A–C). From the several different techniques to form aggregate cultures, the magnetic method was selected, as the PC9 cell line does not easily form aggregate cultures and the magnetic method assures aggregate formation (Figure 3A,B).

Once the aggregates were formed, the presence of ABC transporters was tested. To confirm mRNA levels, qRT-PCR was performed using beta-actin and CK7 as inner controls for total tissue (Figure S4) and epithelial cell specific expression levels (Figure 4A), respectively. Tissue sections prepared from the 3D aggregates were stained for ABCB1 and ABCG2 proteins (Figure 4B). Protein levels were quantified using integrated fluorescence intensity (Figure 4C).

RNA isolation from co-cultures has confirmed that the mRNA pattern of ABCB1 and ABCG2 does not differ significantly between the various cancer cell line containing aggregates either in all cell type containing (Figure S4) or in epithelial cell specific (CK7) calculations (Figure 4A). Different drug transporter expression tendencies became significant at certain protein levels (Figure 4B,C). Significant differences were detected in both ABCB1 and ABCG2 protein levels in KRAS mutant A549 and A549-CR aggregates (Figure 4B,C). Neither ABCB1 nor ABCG2 protein levels changed significantly in the EGFR mutant PC9 and PC9-CR cell lines (Figure 4B,C).

Once the tissue culture system was set up and characterized for further analysis, it was investigated how cisplatin affects drug transporter expression of CR cell lines if treated with carboplatin, paclitaxel, or their combination. These drugs were specifically selected as they are clinically relevant in the treatment of AC-s [43,44,45].

Following exposure to mono- or combination treatment of carboplatin and/or paclitaxel [43,44,45], both ABCB1 and ABCG2 gene expression levels were determined at first in the parental controls (Figure S2). In the KRAS mutant A549 parental cell line, ABCG2 was significantly increased after carboplatin treatment. The ABCB1 mRNA levels did not change. The ABCB1 mRNA levels in the EGFR mutant PC9 parental cell line containing aggregate cultures all treatments induced significant decrease in gene expression. The ABCG2 mRNA levels significantly decreased only after paclitaxel treatment (Figure S2). The background mutation differentially influenced the effect of treatment (Figure S2).

**Figure 5:**
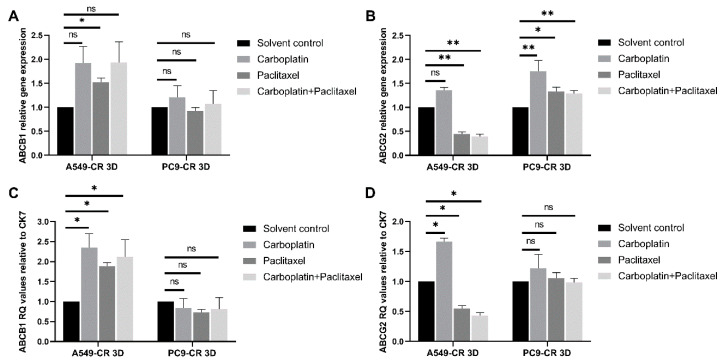
Effect of clinically relevant chemotherapeutic drugs on CR cell line containing 3D aggregates. (**A**) Relative mRNA expression (2^−ddCt^) of ABCB1 drug transporters in cisplatin-resistant AC cell line (A549-CR, PC9-CR) containing 3D aggregate co-cultures. The inner control was beta actin. (**B**) Relative mRNA expression of ABCG2 drug transporters in cisplatin-resistant AC cell line (A549-CR, PC9-CR) containing 3D aggregate co-cultures. The inner control was beta actin. (**C**) Relative mRNA expression (2^−ddCt^) of ABCB1 drug transporters in cisplatin-resistant AC cell line (A549-CR, PC9-CR) containing 3D aggregate co-cultures. The inner control was CK7. (**D**) Relative mRNA expression (2^−ddCt^) of ABCG2 drug transporters in cisplatin-resistant AC cell line (A549-CR, PC9-CR) containing 3D aggregate co-cultures. The inner control was CK7. (“*” *p* < 0.05 and “**” *p* < 0.002).

In the CR cell line containing tissue aggregates the results were rather different (Figure 5A,B).

ABCB1 levels were only increased in the KRAS mutant A549-CR cell containing aggregate cultures after treatment with paclitaxel and had no effect in the PC9-CR containing CR co-cultures (Figure 5A). ABCG2 was significantly decreased after paclitaxel and paclitaxel and carboplatin combination but not carboplatin monotreatment of the KRAS mutant A549-CR cell containing aggregate cultures (Figure 5B). This result indicates that the reducing effect on drug transporter expression is due to paclitaxel and not carboplatin treatment. In the PC9-CR cell, containing aggregates significant increase in ABCG2 expression was detected following carboplatin and paclitaxel (*p* = 0.046), as well as carboplatin and paclitaxel combination treatment (*p* = 0.046) (Figure 5B). To investigate whether the results are associated with the cancer cell lines and not the other cell types in the tissue aggregates, the epithelial marker CK7 was used to detect epithelial cell associated changes in gene expression (Figure 5C,D). The overall pattern of ABCB1 and ABCG2 appeared to be similar to the pattern presented in Figure 5A,B, but with some notable differences. ABCB1 levels increased in the KRAS mutant A549-CR cell containing aggregate cultures after all the applied treatments, while no significant changes were detected in the EGFR mutant PC9-CR containing co-cultures (Figure 5C). ABCG2 expression in the A549-CR containing tissues was significantly induced by carboplatin monotreatment but reduced by paclitaxel mono- and paclitaxel and carboplatin combination treatment, indicating that the drug transporter expression changes were greatly associated with the cancer cells, even in the mixed cell culture. In contrast, ABCG2 in the PC9-CR containing cell co-cultures did not present significant differences following treatment, implicating the role of the other cell types in drug transporter expression. The above experiment has also highlighted the difference induced by background mutation.

### 2.4. WNT Signalling in Lung AC Cell Lines Carrying KRAS or EGFR Mutations

**Figure 6:**
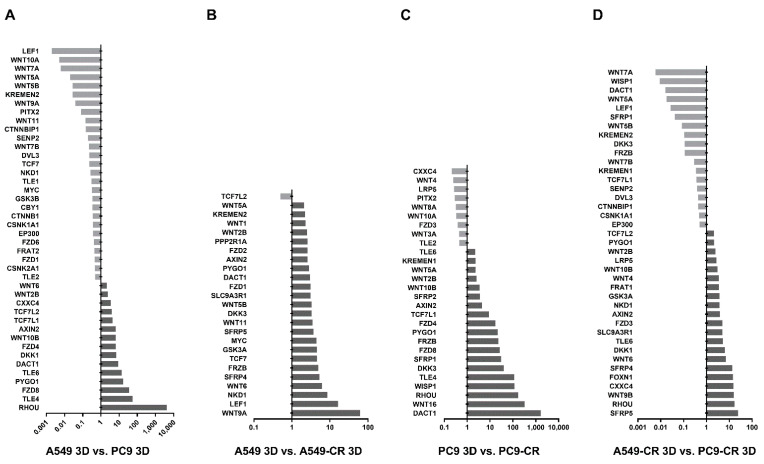
Changes in the WNT microenvironment. (**A**) mRNA expression of WNT signalling pathway genes in A549 3D aggregates relative to PC9 3D aggregates. (**B**) mRNA expression of WNT signalling pathway genes in A549-CR 3D aggregates relative to A549 3D aggregates. (**C**) mRNA expression of WNT signalling pathway genes in PC9-CR 3D aggregates relative to PC9 3D aggregates. (**D**) mRNA expression of WNT signalling pathway genes in A549-CR 3D aggregates relative to PC9-CR 3D aggregates. (The cut-off was at and above a 2-fold increase or decrease in the values detected by the arrays. The full analysis data can be viewed in Figure S3).

Recently, several studies confirmed that the WNT/β-catenin pathway is crucially important in regulating cisplatin resistance [46,47,48]. In the present study, TaqMan arrays were used to investigate whether specific driver mutations and cisplatin resistance can induce alterations in the WNT microenvironment (Figure 6A–D). Three-dimensional aggregate cultures containing A549-CR or parental A549 cells; PC9-CR or PC9 parental cells were assembled (Figure 6A–D).

Analysis of the KRAS mutant and the EGFR mutant cell line containing aggregate cultures revealed that the KRAS mutant A549 cells express Ras Homolog Family Member U (RHOU) nearly a thousand-fold higher than PC9 cell containing 3D aggregate cultures (Figure 6A). In contrast, the EGFR mutant PC9 cells had higher mRNA levels in lymphoid enhancer-binding factor 1 (LEF1), Kringle Containing Transmembrane Protein 2 (KREMEN2), WNT1-inducible-signalling pathway protein 1 (WISP1) and several WNT ligands including WNT4, 5A, 6, 9A and 10B (Figure 6A). Comparing the mRNA levels of 3D aggregates containing A549-CR with parental A549 cells, the expression of three genes increased dramatically: LEF1, Naked Cuticle 1 (NKD1) and WNT ligand 9A (WNT9A) (Figure 6B). Similar comparison of gene transcription in PC9-CR and the parental cell control PC9 containing 3D aggregates highlighted the differential expression of Dishevelled Binding Antagonist of Beta-Catenin 1 (DACT1), RHOU, Transducin-like enhancer protein 4 (TLE4), WISP1 and WNT ligand 16 (WNT16) (Figure 6C). Based on the above data, both the presence of different driver mutations and cisplatin affects and modulates WNT signalling at the ligand and signal transduction level. We can also determine that cisplatin treatment makes the cancer cell lines increasingly similar, rather than different, as far as the WNT signalling microenvironment is concerned (Figure S3).

## 3. Discussion

A major challenge in cancer chemotherapy is MDR. MDR may occur naturally but acquiring MDR during chemotherapy is more frequent [49,50]. Although current laboratory studies and clinical trials have identified highly specific drugs for targeted and personalized therapy, effective clinical intervention to overcome MDR in cases and mostly in advanced stages of the disease where chemotherapy is irreplaceable is still missing [49].

In the present study, we used cisplatin and analysed the molecular microenvironment in the presence of KRAS and EGFR mutation. Compared to normal lung epithelial cells, both AC cell lines had higher ABCB1 and ABCG2 expression than the normal control. However, in the KRAS mutant A549 cell line both ABCB1 and ABCG2 levels were higher than in the EGFR mutant PC9 cell line, indicating that the mutational background matters when it comes to drug transporter expression. Additionally, our data show that chronic exposure to cisplatin treatment can alter gene expression levels of ABC transporters. Both expression and activity of ABCB1 and ABCG2 drug transporters altered depending on the mutation background (KRAS or EGFR).

To increase the similarity of our culture system to the microenvironment of the human lung, the KRAS and EGFR mutant cells (A549, PC9) and cisplatin resistant (A549-CR, PC9-CR) cells were placed into 3D aggregate co-cultures to perform clinically more relevant experiments. Although cisplatin is one of the oldest of cancer drugs, it is frequently used in combination therapy [45]. As cisplatin is highly toxic and not always tolerated by the patient, combination treatments often contain or changed to another platinum-based drug, carboplatin. Carboplatin is used in combination with paclitaxel, vinorelbine or gemcitabine depending on the mutation background of the patient. Using the 3D aggregate cultures, we determined that the use of cisplatin therapy—mostly due to its higher efficacy—before changing to carboplatin or other drugs or drug combinations can significantly increase the mRNA expression levels of ABCB1 (or PGP), the well-known MDR transporter [51]. ABCB1 relieves cancer cells from several chemotherapeutic drugs, including vinca alkaloids, anthracyclines, taxanes, and notably paclitaxel. As paclitaxel is an essential part of the treatment protocol in advanced lung AC [43,44,45,52,53,54], ABCB1 levels are potential indicators of therapy response [55,56]. In 3D co-cultures of cisplatin—resistant KRAS mutant A549-CR cells ABCB1 mRNA levels increased following treatment with carboplatin or paclitaxel in mono-, or in combination treatment. Meanwhile, ABCG2 levels were reduced (Figure 5C,D) signifying the reliable testing potential of a 3D co-culture.

Theoretically, and based on the above data, inhibition of ABCB1 should improve treatment efficacy. It is not surprising that pharmaceutical research has started to focus on the inhibition of ABC transporters. Using Tariquidar (a potent ABCB1-antagonist) has been tested in combination with carboplatin/paclitaxel in phase III trials in NSCLC patients, based on the presumption that the inhibitor is going to increase the effect of cancer therapy. Unfortunately, such trials had to be discontinued due to chemotherapy-related toxicity in the tariquidar arm [57]. Other ongoing or completed clinical studies with tariquidar, paclitaxel, docetaxel, carboplatin and cisplatin suggest that therapeutic modulation of the ABC transporter activity might be beneficial for some LC patients in the future [57,58], but the cancer cell specificity of such drugs has to be improved.

Although, cisplatin is not a substrate of ABCB1 or ABCG2, resistance to cisplatin changes the expression of both transporters [53,56,59]. As such, if a patient can no longer tolerate cisplatin mono- or combination therapy, drug efficacy may be reduced using a second-line drug as a result of preceding administration of cisplatin.

The WNT pathways are particularly important in the regulation of ABC transporter expression [60], as the TCF/LEF (T-cell specific transcription factor/lymphoid enhancer binding factor) beta-catenin dependent WNT signalling activates the ABCB1 promoter and gene transcription leading to increased ABCB1 levels [20,21]. Consequently, modulation of the WNT pathway can affect chemoresistance [22,23]. The highly complex and evolutionarily conserved WNT signalling pathway [60,61] controls many developmental and tissue maintenance events. Previous studies have shown that the WNT pathway, although rarely mutated, is differentially regulated in LC subtypes [60,61]. In the present study, we identified that specific background mutations are associated with differences in WNT gene expression that also affect the response to cisplatin. RHOU which belongs to the Rho family of GTPases and affects cytoskeletal organization, cell proliferation, cell morphology and adhesion, therefore, is highly important in regulating metastasis [62]. RHOU levels were significantly (around 10,000-fold) higher in the KRAS mutant A549 cells than in the EGFR mutant PC9 cells. In contrast, the PC9 cell line overexpressed an array of WNT ligands, including WNT1, WNT7a, WNT7b and WNT9a ligands which have already been reported in lung ACs [60,63]. WNT9a might be particularly interesting as this ligand binds to members of those frizzled family of seven transmembrane receptors that function in the canonical WNT/beta-catenin signalling pathway [63,64].

Prolonged treatment of the EGFR mutant PC9 cells with cisplatin resulted in upregulation of some important genes controlling the beta-catenin dependent WNT pathway. One of these molecules was DACT1 (homologue of Dapper), that is defined as a Dishevelled (DVL)-associated antagonist of the JNK (C-Jun N-terminal kinase) and WNT/beta-catenin pathways [65,66]. In several studies, low levels of DACT1 were described in NSCLC, while its over-expression has also been documented in other cancer types [64,66,67,68]. All in all, the dysregulation of DACT1 is associated with poor prognosis [69]; therefore, DACT1 is under investigation as a potential therapeutic target [66,68]. The parallel increase in WNT16, which advances malignancies by counteracting cell death was recently studied by neutralizing anti-WNT16 antibody, co-administered with classical chemotherapy to increase patient survival [70]. Increased RHOU expression was detected in the EGFR PC9-CR cells [62], while the well-known oncogene in lung and colorectal cancer TLE4 was also induced by cisplatin in the PC9-CR cells [71,72,73]. The similarly upregulated WISP1 has already been reported to contribute to the toxicity of platinum-based chemotherapy in LC [74,75]. The cisplatin induced modification of the WNT microenvironment in the PC9-CR cells indicates that platinum-based chemotherapy may alter individual patient response. In the KRAS mutant cells, the beta-catenin WNT pathway target lymphoid enhancer-binding factor 1 (LEF1) transcription factor TCF/LEF signalling was drastically upregulated [75,76] by cisplatin. NKD1, a well-known negative feedback regulator of the canonical WNT pathway [77,78,79], was also upregulated in the A549-CR cell line, indicating an attempted control over beta-catenin dependent signal regulation. Comparison of the two CR cell lines have resulted some additional details. While in the KRAS mutant A549-CR cell line, soluble inhibitors (e.g., soluble frizzled receptors) were more highly expressed, the EGFR mutant PC9-CR cells expressed an array of WNT ligands, DACT1, WISP1 and KREMEN-2 co-receptor higher than the A549-CR-s. It indicates that, although the background mutation modulates the response to cisplatin by the activation of different WNT signalling pathways, the actual outcome is only marginally different, as drug transporters, especially ABCB1 expression, increases. As the WNT pathway activation of the EGFR mutant PC9 cell line involves molecules that are under direct investigation as therapeutic targets (e.g., DACT1 and WNT16), in patients with the EGFR mutant, AC might have additional therapeutic targets that are potentially safer and more effective than directly inhibiting the ABCB1 transporter.

## 4. Materials and Methods

### 4.1. Human Lung Cancer Tissues

Twelve primary human lung AC samples (Table 1) were obtained after surgery and assessed by a certified lung pathologist at the University of Pecs, Hungary. None of the patients were pre-treated with chemotherapeutic drugs before surgery. Patients provided written informed consent and the study was approved by the Ethical Committee of the University of Pecs and the Medical Research Council of Hungary (ETT-TUKEB 366/2015). All collected samples were treated anonymously following the guidelines and regulations of the 1975 Helsinki Declaration.

### 4.2. Cell Cultures

Human NSCLC AC cell lines A549 (KRAS mutant) and PC9 (EGFR mutant: DelE746A750) (American Type Cell Culture Collection, Rockville, MD, USA) were used in the experiments. A549 cells were cultured in complete Dulbecco’s Modified Eagle’s Medium (DMEM) (Lonza, Walkersville, MD, USA), supplemented with 10% foetal calf serum (FCS), 3% Penicillin/Streptomycin, 2% L-Glutamine, 1% non-essential amino acid, 1% 4-(2-hydroxyethyl)-1-piperazineethanesulfonic acid (HEPES) buffer and 1% β-mercaptoethanol. PC9 cells were cultured in Rosewall Park Memorial Institute 1640 Medium (RPMI) (Thermo Fisher Scientific, Waltham, USA) supplemented with 10% FCS, 3% Penicillin/Streptomycin, 2% L-Glutamine. Primary small airway epithelial cells (SAEC) were purchased from Lonza (Lonza, Walkersville, MD, USA) and cultured in Small Airway Growth Medium (Lonza, Walkersville, MD, USA). Normal human lung fibroblasts cells (NHLF) were cultured in Fibroblast Growth Medium (FGM-2) and Primary Umbilical Vein Endothelial Cells; Normal, Human (HUVEC) (American Type Cell Culture Collection, Rockville, MD, USA, ATCC^®^ PCS-100-010™), were maintained in F-12K Medium (Kaighn’s Modification of Ham’s F-12 Medium) (ATCC^®^ 30-2004™), respectively. All types of cells were cultured at 37 °C in humidified atmosphere containing 5% CO_2_. Trypan blue dye exclusion test was used to assess cell viability [80].

### 4.3. Three Dimensional (3D) Aggregate Cultures

3D aggregates consisted of NHLF, HUVEC and A549 or PC9 cells at 4:3:3 ratio. The aggregates were prepared by a commercially available magnetic cell levitation technique using the 96 Well Bioprinting Kit and the 96 Well BiOAssay™ Kit provided by Greiner Bio-One Ltd., (Kremsmünster, Austria). The cells were incubated with NanoShuttle^TM^-PL at 4°C for 4 h, respectively (A549, A549-CR, PC9, PC9-CR, NHLF, HUVEC). The magnetised cells were detached, counted, and dispensed in a cell-repellent plate. Cell-repellent plate was placed on the holding drive to aggregate the cells and incubated at 37 °C for 15 min. The aggregates were suitable for gene expression testing and immune-histochemical staining of drug transporters.

### 4.4. Immune-Histochemical Staining of Drug Transporters

The 3D aggregates were sectioned using a Leica CM1950 cryostat (Leica, Wetzlar, Germany), then fixed and stained using a routine IHC staining procedure in a Vision Biosystems bond™ automated immune-stainer (Leica, Wetzlar, Germany). Anti-human ABCG2 mouse monoclonal antibody (CD338, clone 5D3, BD Biosciences, San Jose, CA, USA) and anti-human ABCB1 rabbit monoclonal antibody (clone D3H1Q, Cell Signaling Technology, Danvers, MA, USA) were used as primary antibodies, both in 1:50 dilution. EpCAM (CD326, 323/A3, MA5-12436, ThermoFisher Scientific, MA, USA) unlabelled mouse monoclonal antibody in 1:50 and Cytokeratin 5 polyclonal rabbit (SC-66856, Santa Cruz Biotechnology, Dallas, TX, USA) in 1:100 dilution was used. The secondary antibody was a goat anti-rabbit IgG antibody (Alexa Fluor^®^ 647) (ab150087) (1:2000) (Abcam Plc, Cambridge, United Kingdom) and the anti-mouse antibody was an Alexa Fluor^®^ 488 conjugated IgG (Thermo Fisher Scientific, Waltham, USA) (dilution 1:200). Nuclei were counterstained with Dapiprazole hydrochloride (DAPI) (ab142859) (1:1000) (Abcam Plc, Cambridge, United Kingdom). Images were acquired applying Nikon Eclipse Ti-U microscope (Nikon GmbH CEE, Vienna, Austria) equipped with CCD camera (AndorZyla 5.5), ImageJ (Java) applied for densitometry. Drug transporter protein intensity quantification was normalized to the respective epithelial marker intensity.

### 4.5. RNA Isolation, cDNA Synthesis and qRT-PCR

Cell cultures were harvested in RA1 Buffer Solution then RNA isolation was performed using NucleospinII RNA isolation kit according to the manufacturers’ protocol (Macherey-Nagel, Düren, Germany). Total RNA from frozen human lung samples was isolated using TRI Reagent (Molecular Research Center, Cincinnati, OH, USA) with an additional on-column DNaseI (Sigma-Aldrich, St. Louis, MO, USA) treatment. RNA concentration was measured using Nanodrop technology (Thermo Fisher Scientific, Waltham, MA, USA).

cDNA synthesis was performed using random hexamer primers of the high-capacity RNA to cDNA kit (Thermo Fisher Scientific, Waltham, MA, USA) according to the manufacturers’ protocol. SYBRGreen (Roche, Basel, Switzerland) real-time qRT-PCR reaction using sequence specific primers (Table 2) was set up using Applied Biosystems 7500 Real-Time PCR System (Thermo Fisher Scientific, Waltham, MA, USA). The relative quantities of different drug-transporters were calculated using the 2^−ddCt^ method. The inner control was beta-actin for each sample.

### 4.6. Functionality Test of ABC Drug Transporters

Wild-type and cisplatin-resistant (CR) A549 and PC9 cells were washed with PBS then trypsinized. Cells were counted, and sorted into FACS tubes, 100000 cells into each and in triplicates. MultiDrugQuantTM Kit (SOLVO Biotechnology, Szeged, Hungary) and flow-cytometry were used to measure the functional activity of ABCB1 and ABCG2 transporters. BD FACScanto II (BD Biosciences, San Jose, CA, USA) was used for flow-cytometry with a BD FACSDiva V5.1 software (BD Biosciences, San Jose, CA, USA). The results were calculated as multidrug resistance (MDR) activity factor values (MAF) according to the instructions of the manufacturer. The calculations were performed using the median of triplicates. MAF values at 20 and above are regarded as representing functionally active drug transporter proteins.

### 4.7. Drugs and Reagents

Drugs including cisplatin, carboplatin, and paclitaxel were purchased from Selleckchem (Houston, TX, USA). For the generation of CR NSCLC cell lines, the starting concentration of cisplatin in A549 cell cultures was 1 µM, while the final concentration reached 12 µM within 3 weeks. In the PC9 cell cultures starting concentration of cisplatin was 0.67 µM that was increased to 4.67 µM within 3 weeks. Carboplatin and paclitaxel treatment were applied for 24 h in 100 µM and in 0.002 µM concentrations, respectively.

### 4.8. WNT Signalling Arrays

Relative mRNA expression (2^−ddCt^) of WNT signalling pathway genes in A549, A549-CR and PC9, PC9-CR in 3D aggregates was assessed using Applied Biosystems™ TaqMan™ Array, Human WNT Pathway, Fast 96-well (Thermo Fisher Scientific Inc. TMO, Waltham, MA, USA).

### 4.9. Statistical Analysis

Statistical analysis was performed using the SPSS 26.0 package for Windows (IBM SPSS Statistics, Chicago, IL, USA). Normal distribution was tested using the Kolmogorov–Smirnov test. The differences between 2 independent groups were calculated using the Mann–Whitney-U test as non-parametric calculation. For normal distribution, the independent-samples t-test and Kruskal–Wallis test were performed. Results were considered statistically significant if *p* < 0.05.

## Figures and Tables

**Table 1:** Patient data.

Number	Mutation	Histology	T	N	M	Age	Gender
1	EGFR/KRAS WT	AC	T2	N1	Mx	65	F
2	EGFR/KRAS WT	AC	T1	N1	Mx	69	M
3	EGFR MUTANT	AC	T2b	N1	Mx	73	F
4	EGFR MUTANT	AC	T1	N1	Mx	60	M
5	KRAS MUTANT	AC	T1	N1b	Mx	65	M
6	KRAS MUTANT	AC	T2b	N2	M0	62	F
7	KRAS MUTANT	AC	T1	N2	Mx	51	F
8	KRAS MUTANT	AC	T3	N2	Mx	57	F
9	KRAS MUTANT	AC	T2	N0	Mx	72	M
10	KRAS MUTANT	AC	T2	N2	Mx	62	M
11	KRAS MUTANT	AC	T2	N2	Mx	68	M
12	KRAS MUTANT	AC	T2	N1	Mx	59	M

**Table 2:** Primer sequences.

Target	Forward Primer	Reverse Primer
human beta-actin	5′-GCGCGGCTACAGCTTCA-3′	5′-CTTAATGTCACGCACGATTTCC-3′
human ABCB1	5′-GCAGCTGGAAGACAAATACACAA-3′	5′-CCCAACATCGTGCACATCA-3′
human ABCG2	5′-AACCTGGTCTCAACGCCATC-3′	5′-GTCGCGGTGCTCCATTTATC-3′
human ABCC1	5′-GCTGGAGTGTGTGGGCAACT-3′	5′-CTGAGGCGTTGCCTGGAGAT-3′
human ABCC2	5′-GCAAACTGTTCTGGTGTGGGA-3′	5′-CCAGCTCTATGGCTGCTAGA-3′
human ABCC6	5′-GAATGGCCTGGTGTTTGCAG-3′	5′-CAGTTGCGAACAACCCACTG-3′
human ABCC10	5′-AACGCTTTGCCAACAAGACA-3′	5′-CCAGCACCCGGTCTGAGTT-3′
human CK7	5′-AGGATGTGGATGCTGCCTAC-3′	5′-GGGACTGCAGCTCTGTCAAC-3′

## Data Availability

Data is contained within the article or Supplementary Material.

## Supplementary Materials

The following are available online at https://www.mdpi.com/article/10.3390/ijms22105384/s1, Figure S1: Gene expression pattern of ABCC1, ABCC2, ABCC6, ABCC10 in 2D cell cultures; Figure S2: Effect of clinically relevant chemotherapeutic drugs on parental cell line containing 3D aggregates; Figure S3: Changes in WNT signalling pathway activation; Figure S4: Relative mRNA expression (2^−ddCt^) of drug transporters in 3D aggregates.

## Abbreviations

ABC transporter	ATP Binding Cassette Family of transporters
ABCB1	ATP Binding Cassette Subfamily B (MDR/TAP) Member 1
ABCC1	ATP Binding Cassette Subfamily C Member 1
ABCC2	ATP Binding Cassette Subfamily C Member 2
ABCC6	ATP Binding Cassette Subfamily C Member 6
ABCC10	ATP Binding Cassette Subfamily C Member 10
ABCG2	ATP Binding Cassette Subfamily G (BCRP) Member 2
AC	adenocarcinoma
AE	alveolar epithelium
ALK	anaplastic lymphoma kinase
BRAF	B-Raf Proto-Oncogene, Serine/Threonine Kinase
CR	cisplatin-resistant
DACT1	Dishevelled Binding Antagonist of Beta Catenin 1
DMEM	Dulbecco’s Modified Eagle’s Medium
DVL	Dishevelled protein
EGFR	epidermal growth factor receptor
F-12K	Kaighn’s Modification of Ham’s F-12 Medium
FCS	foetal calf serum
FGM-2	Fibroblast Growth Medium
FZ	Frizzled protein
HEPES	4-(2- hydroxyethyl)-1-piperazineethanesulfonic acid
HUVEC	Primary Umbilical Vein Endothelial Cells; Normal, Human
JNK	C-Jun N-terminal kinase
KRAS	Kirsten rat sarcoma viral oncogene homolog
KREMEN2	Kringle Containing Transmembrane Protein 2
LC	lung cancer
LCC	large cell carcinoma
LEF1	lymphoid enhancer-binding factor 1
MAF	multidrug resistance activity factor
MDR	multidrug resistance
NHLF	normal human lung fibroblasts
NKD1	Naked Cuticle 1
NSCLC	non-small cell lung cancer
PDL1	programmed cell death ligand1
PE	primordial epithelium
RHOU	Ras Homolog Family Member U
ROS1	proto-oncogene 1, receptor tyrosine kinase
RPMI	Rosewall Park Memorial Institute 1640 Medium
SAEC	small airway epithelial cell
SCC	squamous cell carcinoma
SCLC	small cell lung cancer
TCF/LEF	T-cell specific transcription factor/lymphoid enhancer binding factor
TKI	tyrosine kinase inhibitor
TLE4	Transducin-like enhancer protein 4
WISP1	WNT1-inducible-signaling pathway protein 1
WNT	Wingless-Type MMTV Integration Site Family
WT	wild type
