# Supplementary material for: KRAS and EGFR Mutations Differentially Alter ABC Drug Transporter Expression in Cisplatin-Resistant Non-Small Cell Lung Cancer
Source: Int J Mol Sci. 2021 May 20;22(10):5384. doi: 10.3390/ijms22105384 (PMC8160643; doi:10.3390/ijms22105384)
Supplement: Supplementary file 1 [file ijms-22-05384-s001.zip › ijms-1210896-supplementary.pdf]

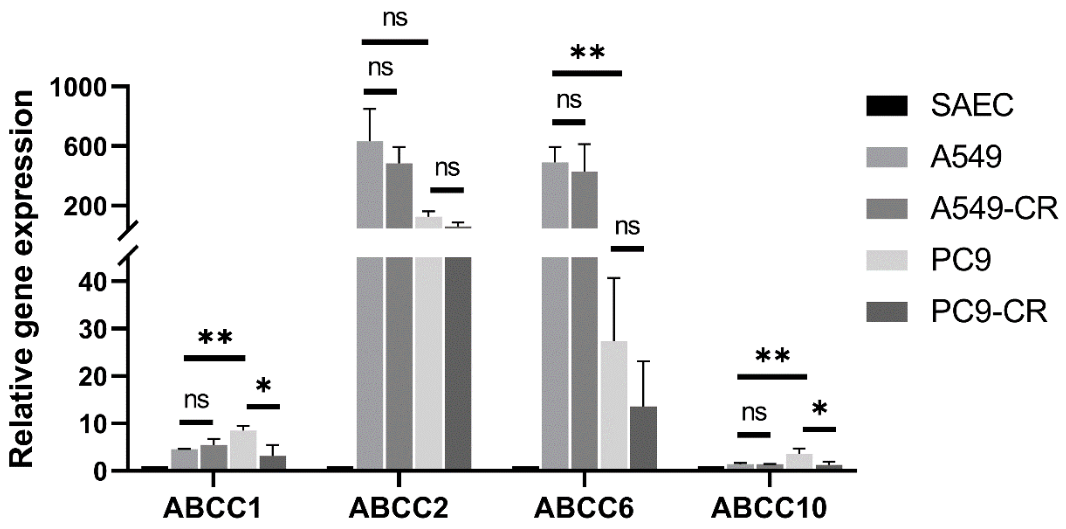

Supplementary Figure 1. Gene expression pattern of ABCC1, ABCC2, ABCC6, ABCC10 in 2D cell cultures

Relative mRNA expression (2^{-ddCt}) of ABCC1, ABCC2, ABCC6 and ABCC10 drug transporters in solvent control parental and cisplatin-resistant adenocarcinoma cell lines (A549, A549-CR, PC9, PC9-CR) compared to normal, primary human SAEC. The graph shows the mean and SD of biological repeats (n=3).

A

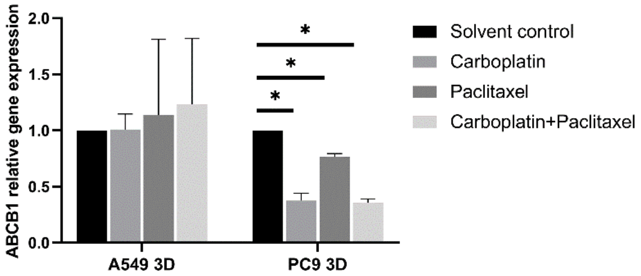

B

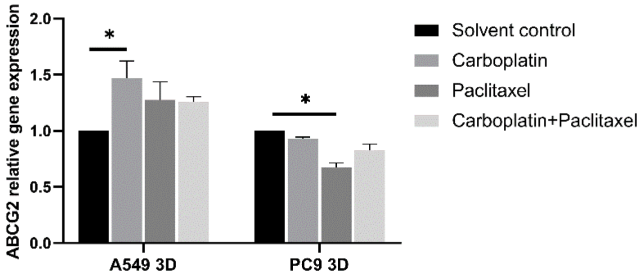

Supplementary Figure 2. Effect of clinically relevant chemotherapeutic drugs on parental cell line containing 3D aggregates. (a) Relative mRNA expression (2^{-ddCt}) of ABCB1 drug transporters in solvent control parental AC cell line (A549, PC9) containing 3D aggregate co-cultures. The inner control was beta actin. **(b)** Relative mRNA expression of ABCG2 drug transporters in solvent control parental AC cell line (A549-CR, PC9-CR) containing 3D aggregate co-cultures. The inner control was beta actin.

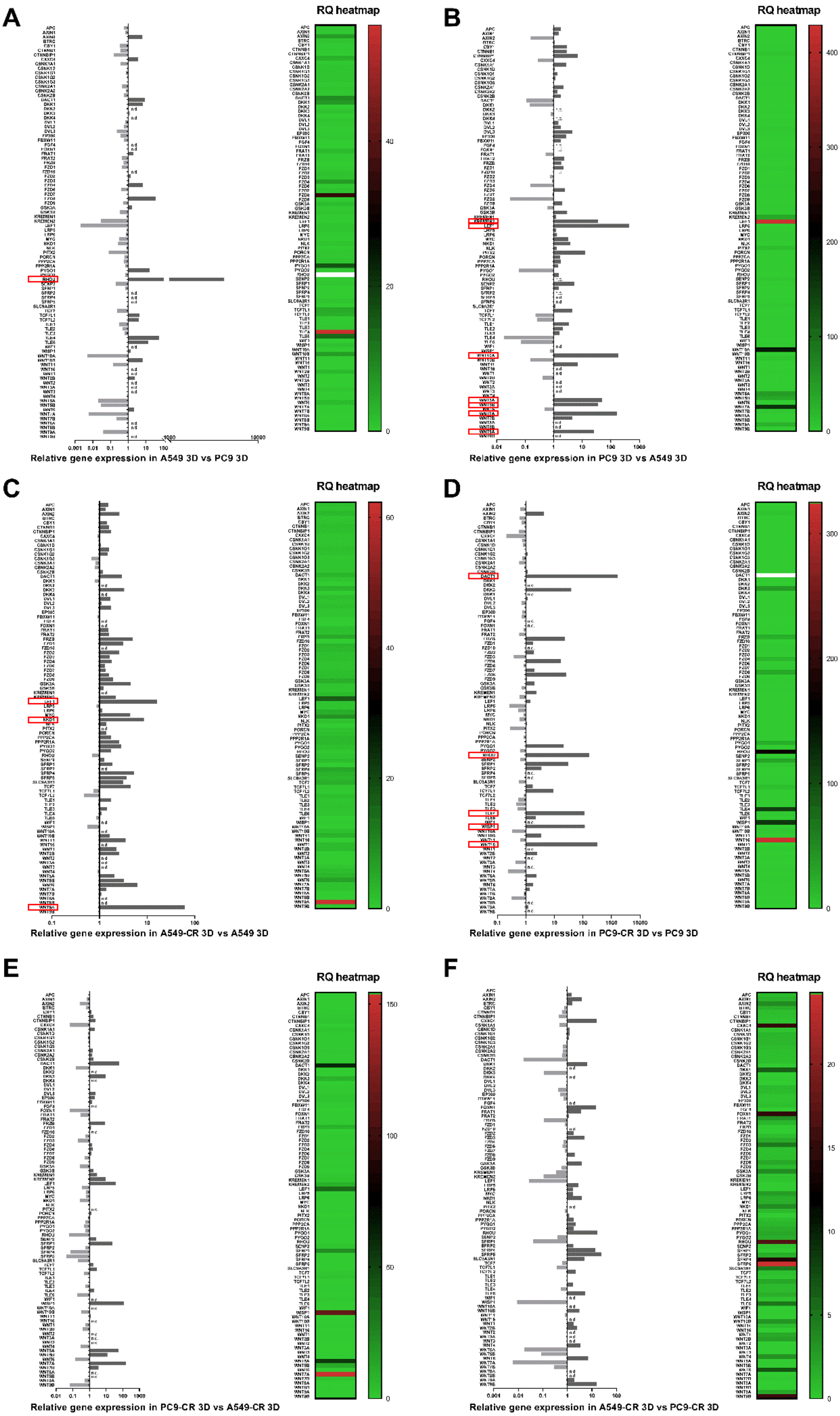
A
RQ heatmap
Relative gene expression in A549 3D vs PC9 3D
B
RQ heatmap
Relative gene expression in PC9 3D vs A549 3D
C
RQ heatmap
Relative gene expression in A549-CR 3D vs A549 3D
D
RQ heatmap
Relative gene expression in PC9-CR 3D vs PC9 3D
E
RQ heatmap
Relative gene expression in PC9-CR 3D vs A549-CR 3D
F
RQ heatmap
Relative gene expression in A549-CR 3D vs PC9-CR 3D

Supplementary Figure 3. Changes in Wnt signalling pathway activation. (a) Relative mRNA expression (2^{-ddCt}) of Wnt signalling pathway genes in A549 3D aggregates relative to PC9 3D aggregates. **(b)** Relative mRNA expression (2^{-ddCt}) of Wnt signalling pathway genes in PC9 3D aggregates relative to A549 3D aggregates. **(c)** Relative mRNA expression (2^{-ddCt}) of Wnt signalling pathway genes in A549-CR 3D aggregates relative to A549 3D aggregates. **(d)** Relative mRNA expression (2^{-ddCt}) of Wnt signalling pathway genes in PC9-CR 3D aggregates relative to PC9 3D aggregates. **(e)** Relative mRNA expression (2^{-ddCt}) of Wnt signalling pathway genes in PC9-CR 3D aggregates relative to A549-CR 3D aggregates. **(f)** Relative mRNA expression (2^{-ddCt}) of Wnt signalling pathway genes in A549-CR 3D aggregates relative to PC9-CR 3D aggregates.

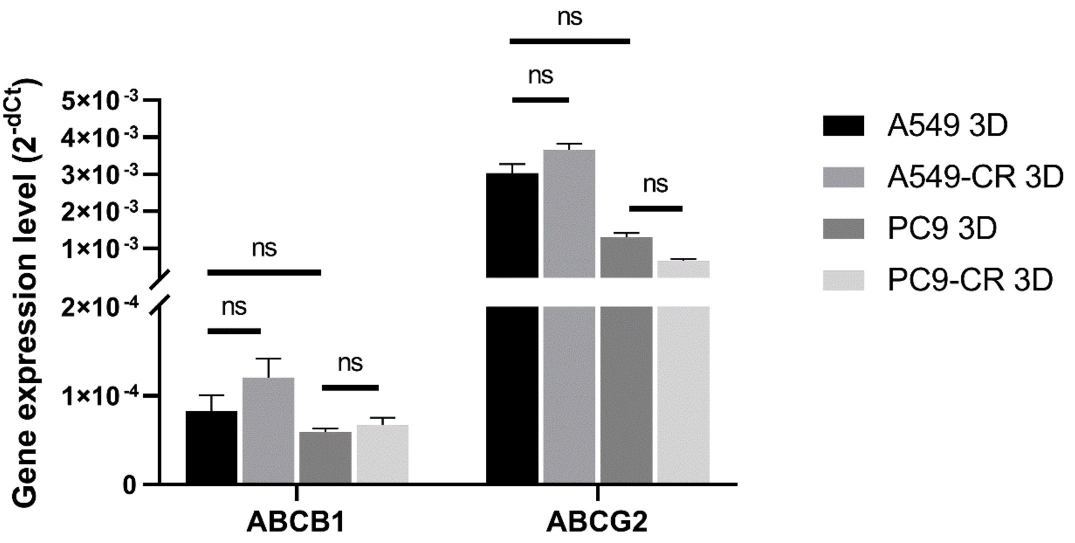

Supplementary Figure 4. Relative mRNA expression (2^{-ddCt}) of drug transporters in 3D aggregates. Relative mRNA expression (2^{-ddCt}) of ABCB1 and ABCG2 drug transporters in parental (A549, PC9) and cisplatin-resistant AC cell line (A549-CR, PC9-CR) containing 3D aggregate co-cultures. The inner control was beta-actin. Biological repeats n=3.
